# Late recovery from unconsciousness in a patient with severe posterior reversible encephalopathy syndrome

**DOI:** 10.1002/ccr3.1740

**Published:** 2018-07-30

**Authors:** Shiori Ogura, Hiromichi Narumiya, Ryoji Iiduka, Yoshinari Nagakane

**Affiliations:** ^1^ Division of Neurology Japanese Red Cross Kyoto Daini Hospital Kyoto Japan; ^2^ Emergency and Critical Care Center Japanese Red Cross Kyoto Daini Hospital Kyoto Japan

**Keywords:** cytotoxic edema, posterior reversible encephalopathy syndrome, prolonged unconsciousness, vasogenic edema

## Abstract

This study describes a patient case presenting with severe posterior reversible encephalopathy syndrome (PRES) who needed 3 months to recover impaired consciousness. We discuss the protracted time course needed to deal with severe PRES cases. Positive prognoses can emerge from these situations if treatment is prompt and precise.

## 1 INTRODUCTION

Posterior reversible encephalopathy syndrome (PRES) is characterized by temporary vasogenic edema and has varied symptoms, but its symptoms disappear in about a week. A man with disturbed consciousness was brought to our hospital. He had severe hypertension and hyperuremia. Magnetic resonance imaging showed vasogenic edema changes over a wide area; this indicated PRES. He showed some indicators of poor prognosis, but he gained consciousness after more than 3 months and another 2 months to experience full symptom resolution. To the best of our knowledge, this is the first case in which a symptom of PRES resolved after a long period. Appropriate and prompt treatment can aid in diagnosing severe PRES even in cases that resolve after a long time. For such cases, the patient's progress should be monitored for more than 3 months.

Posterior reversible encephalopathy syndrome is a clinical disease concept first advocated by Hinchey et al^1^ PRES is typically characterized by reversible vasogenic edema, predominantly in the posterior parietal and occipital lobes. Magnetic resonance imaging (MRI) is the key method for diagnosing PRES. T2‐weighted images and fluid‐attenuated inversion recovery (FLAIR) sequences visualize hyperintense signal alteration in the PRES lesion.^2^ However, the pathophysiology of PRES has yet to be clarified. Popular causal factors include toxic agents, hypertension, eclampsia, infection, and autoimmune disease.^3^ Clinical symptoms also vary among patients. Frequently, impaired consciousness and seizures can emerge along with headaches, visual abnormalities, nausea, etc.^3^, ^4^ Several reports have observed symptom resolution within a week, and imaging abnormalities are alleviated in most cases.^5^ However, it has been recently shown that among patients with severe PRES, only about half fully recover, and mortality rates have been documented at 15%.^6^ In a case that suggests a poor and long‐term prognosis, the symptoms do not fully resolve in just a few days.^7^ However, when promptly recognized and treated, symptoms and radiologic abnormalities can be completely reversed in severe PRES. Nevertheless, when unrecognized, conditions can progress toward ischemia, a massive infarction, or even death.^5^, ^6^, ^7^ The present case describes a patient with severe PRES who needed 3 months to recover impaired consciousness, and full symptom resolution was obtained at 5 months.

## 2 CASE

A 70‐year‐old man, with no previous medical history, was admitted to our hospital due to impaired consciousness. On arrival at our emergency department, his airway was patent and the respiratory rate was 30 times/min. His eyes were open but appearing horizontal nystagmus. The oculocephalic reflex was negative at both sides. He did not react to any induced, painful stimulation and flexed his limbs involuntarily. His elbow and knee joints were spastic, but he did not have obvious paralysis or any sensory disturbance. His blood pressure was 230/165 mm Hg upon admission. A blood chemistry analysis showed hypernatremia (160 mEq/L) and severe kidney injury with azotemia (blood urea nitrogen was 198.9 mg/dL). An arterial blood gas analysis showed metabolic acidosis with respiratory compensation. An echocardiogram revealed concentric hypertrophy of the left ventricle with normal wall motion with hypovolemia. A cranial computed tomography (CT) image obtained on the day of admission demonstrated abnormal low‐density areas in the basal ganglia, midbrain, thalamus, and both sides of the cerebral white matter. He was treated with drip infusion of a calcium channel blocker, and his blood pressure was controlled to 149/88 mm Hg within 5 hours.

**Figure 1:**
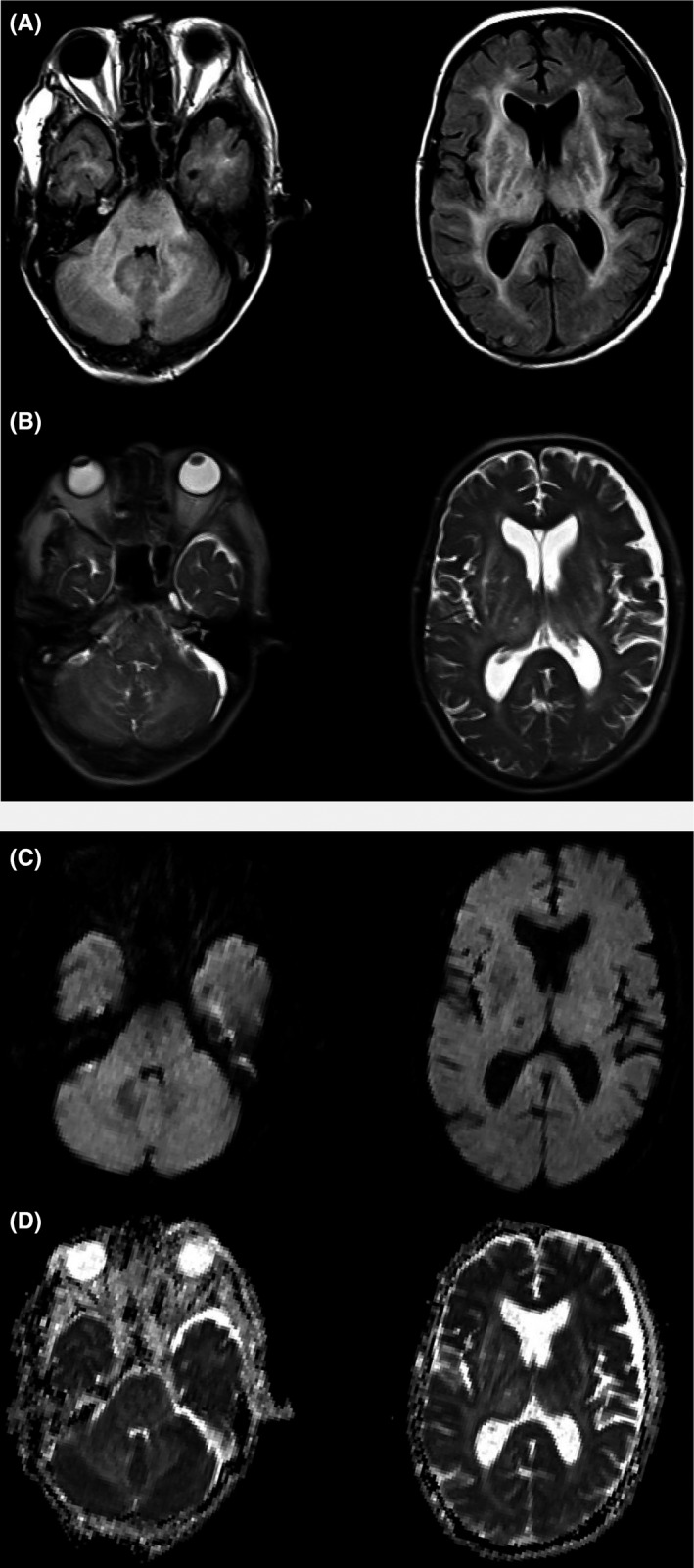
Brain magnetic resonance imaging (MRI) on day 2. T2‐weighted images (A) showed high‐intensity areas in the brainstem, cerebellum, and cerebral white matter (symmetrically). Fluid‐attenuated inversion recovery (FLAIR)images (B) showed the same high‐intensity areas as observed on the T2‐weighted images. There were no apparent abnormally intense areas indicating an acute brain infarction on diffusion‐weighted imaging (DWI) (C) and apparent diffusion coefficient (ADC) maps (D)

**Figure 2:**
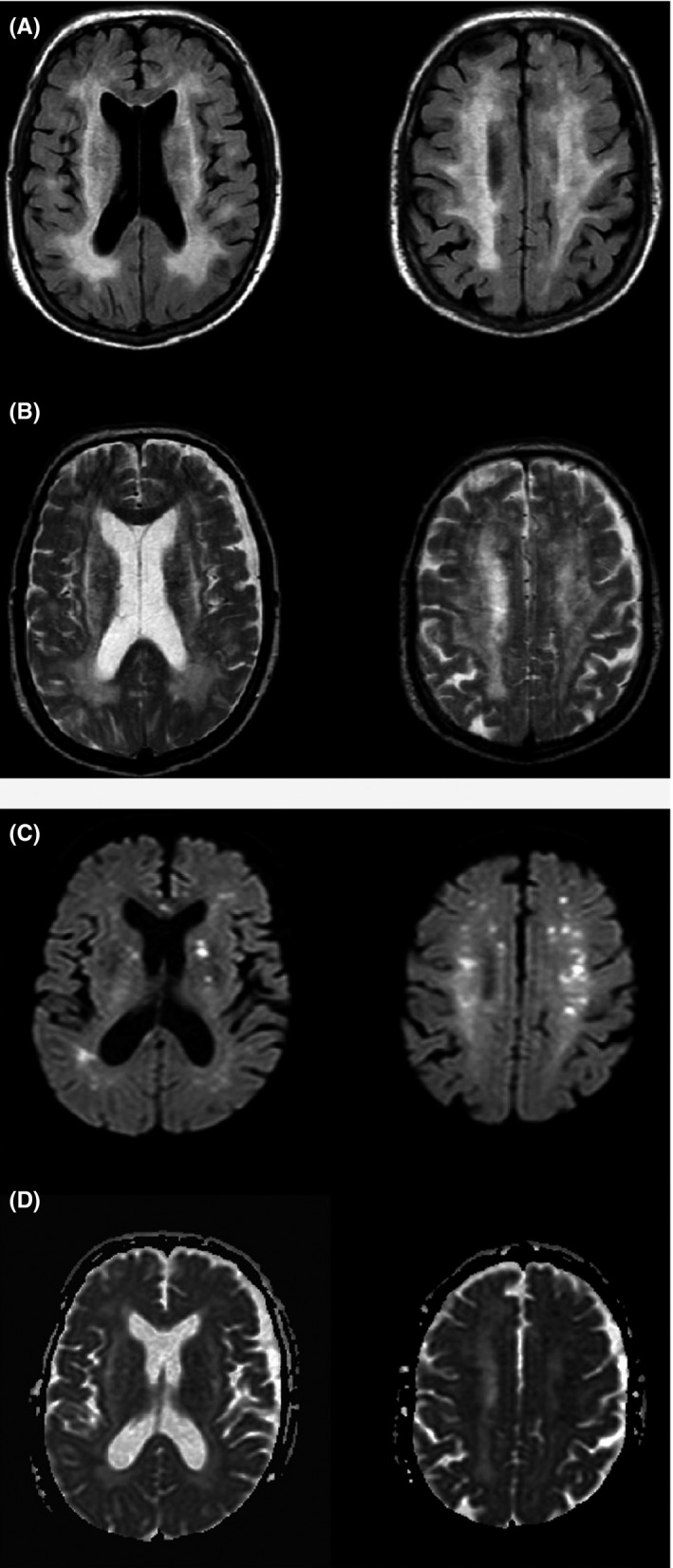
Follow‐up magnetic resonance imaging (MRI) on day 10. Increased signal intensities in T2‐weighted images (A) and fluid‐attenuated inversion recovery (FLAIR) images (B) were unchanged. Note the several microhyperintense signal alterations from the bilateral basal ganglia to the cerebral white matter on the diffusion‐weighted imaging (DWI) (C) with decreased signals on the apparent diffusion coefficient (ADC) maps (D)

A brain MRI obtained on day 2 demonstrated hyperintense lesions in the cerebral white matter, cerebellum, and brainstem. These lesions were hyperintense on apparent diffusion coefficient (ADC) maps. Diffusion‐weighted imaging (DWI) indicated no remarkable alteration (Figure 1). On day 3, sodium concentration was normalized, but the patient's consciousness level was E4V2M4 as determined by the Glasgow Coma Scale. We started hemodialysis that same day and continued nine sessions until azotemia recovered. After weaning from hemodialysis, disturbed consciousness persisted. As noted on an electroencephalogram (EEG), slow waves were detected at all channels without any spike waves. In follow‐up MRI performed on day 10, DWI demonstrated several microhyperintense signal alterations in the bilateral basal ganglia and cerebral white matter, showing low signal intensity on the ADC map (Figure 2). These images verified acute brain infarction. T2‐weighted and FLAIR images showed that previous white matter lesions were constant. Given the diffuse and severely abnormal aspects of the white matter and brain ischemia, discontinuation of treatment was discussed. Eventually, we decided to continue treatment. The patient's level of consciousness did not change significantly, and he presented with an incidental, recurrent urinary tract infection and tympanitis. During the seventh week after his admission, the patient was able to nonverbally answer simple questions (eg, via a head nod). His consciousness gradually returned, and within 3 months, he was able to follow commands more easily. The patient had regained almost full consciousness by the time he was transferred to a rehabilitation hospital after 5 months. However, follow‐up MRI performed during this same period did not indicate the significant resolution of hyperintense alteration in the cerebral white matter, cerebellum, and also brainstem.

## 3 DISCUSSION

A prognosis for PRES, in general, is quite positive. Imaging abnormalities tend to resolve within several weeks, and symptoms tend to disappear within a few days to a week.^4^, ^5^ On the other hand, a recent study revealed that among patients with severe PRES, only about half show adequate recovery.^6^ A limited conscious state can be prolonged beyond a week.^5^ The present case demonstrating severe PRES revealed that impaired consciousness persisted for longer than 1 week and significant symptom improvement did not emerge for nearly 3 months.

Various factors, such as sepsis, dementia, and imaging abnormalities (eg, intracerebral hemorrhage, brain infarction, or a wide area of vasogenic edema), are related to neurological outcomes and PRES severity.^5^, ^7^ Other research has shown that factors associated with poor outcomes include hyperglycemia on day 1, required time to control causes of PRES, etiology, and consciousness disorder.^6^, ^8^ It was previously believed that the severity of an imaging abnormality did not necessarily correspond to specific symptoms; however, high DWI signal intensity and normalized ADC values are associated with cerebral infarction. This might represent the earliest sign of nonreversibility, as severe vasogenic edema progresses toward cytotoxic edema.^9^

The ischemic area is generally contained within the vasogenic edematous region and can expand regardless of regional vascular supply.^10^, ^11^ DWI can be reliably used to distinguish vasogenic edema in PRES from cytotoxic edema in cerebral ischemia.^9^ MRI with diffusion‐weighted sequences provides not only a powerful means for diagnosing PRES but also a wealth of prognostic information regarding the patient. The hallmark of a PRES diagnosis is vasogenic edema in the territories of posterior circulation, which can be reliably differentiated from cytotoxic edema in other etiologies. This is done using DWI and calculating the ADC map, which shows elevated ADC values. DWI might show foci of high signal intensity in the cortex that is either undergoing infarction or at high risk of infarction. ADC values in these areas are normal or slightly elevated. This finding might represent an early sign of nonreversibility in PRES, heralding a conversion toward infarction.^9^ The extent of T2 and DWI signal intensity correlates well with outcomes and can help guide more aggressive treatment among severely affected patients. A brain MRI of the present patient demonstrated hyperintense lesions in the cerebral white matter, cerebellum, and brainstem on T2 and diffusion‐weighted images; however, neurological functioning eventually recovered in the present case. Thus, the contribution of MRI for patient prognosis should be reconsidered.

Furthermore, etiological factors relevant to the present case need to be noted.^12^ For instance, cytotoxic edema might have resulted from longer exposure to initial source toxicities, uremia, and hypertension. We promptly controlled the patient's abnormal hypertension and hypernatremia; however, it took longer to eliminate all uremic substances and improve endothelial dysfunction. Furthermore, the severity of MR imaging lesions including ADC values may be an important parameter determining long‐term prognosis.^13^ In this patient, in follow‐up MRI performed on day 10, ADC map showed low signal intensity in the bilateral basal ganglia and cerebral white matter.

The prognosis is mainly determined by the underlying pathology.^14^ Appropriate and prompt treatment can aid in diagnosing severe PRES even in cases that resolve after a long time. Therefore, a significant amount of time was needed to alleviate all of the patient's disturbance of consciousness.

## 4 CONCLUSION

Posterior reversible encephalopathy syndrome is generally considered a less severe disorder, but this is not always the case. The present patient case describes significant neurological lesion manifestation, serious mental disturbances, and several other factors that led to poor recuperation. However, the patient recovered from most symptoms after 3 months. Thus, when treatment is precise and prompt, a patient with severe PRES can be settled, even if recovery is protracted. Future work will be needed for better understand critical PRES prognoses that extend beyond the 90‐day period observed in the present study.

## CONFLICT OF INTEREST

None declared.

## AUTHORSHIP

SO and HN: equally made the substantial contribution to the conception and drafting of the manuscript. RI: helped to draft the manuscript and revising it critically for important intellectual content. YN: made contributions to analysis and interpretation of brain MRI and clinical data. All authors: gave final approval of the version to be submitted and any revised version.
